# Seven-Day vs Four-Day Infusion Set Replacement Interval and Catheter-Related Infections

**DOI:** 10.1001/jamanetworkopen.2025.46398

**Published:** 2025-12-02

**Authors:** Shalini Elangovan, Yiying Cai, Brett G. Mitchell, Nicholas Graves

**Affiliations:** 1 Programme in Health Services Research and Population Health, Duke–NUS Medical School, Singapore; 2 School of Nursing and Health, Avondale University, Cooranbong, New South Wales, Australia; 3 Hunter Medical Research Institute. Newcastle, New South Wales, Australia; 4 Central Coast Local Health District, Gosford, New South Wales, Australia

## Abstract

**Importance:**

Catheter-related bloodstream infections (CRBSIs) are associated with longer hospital stays and increased mortality risks. The Replacement at Standard vs Prolonged Interval (RSVP) trial found that central venous access devices and peripheral arterial catheter infusion set replacement intervals can be extended from 4 to 7 days without increasing infection risk by more than 2%. However, the trial did not consider the full cost and health trade-offs of extending replacement intervals.

**Objective:**

To estimate the changes in total cost and health benefits associated with a decision to adopt 7-day vs standard 4-day replacement intervals via decision-analytic modeling.

**Design, Setting, and Participants:**

In this economic evaluation, a decision tree model was developed on August 26, 2025, from a health care perspective incorporating data from patients in the RSVP randomized clinical trial conducted from 2011 to 2016 across 10 Australian hospitals. Data were analyzed from December 12, 2016, to April 23, 2019.

**Main Outcomes and Measures:**

A probabilistic sensitivity analysis generated a joint distribution of the expected change to cost and effectiveness outcomes. Parameter uncertainty was assessed, a cost effectiveness acceptability curve was plotted, and a value of information analysis was done.

**Results:**

Of 2941 patients from the RSVP trial (median age, 59.0 years; range, 47-68 years), 62.9% were male. Of patients, 62.6% were admitted to intensive care, and common specialties were medical, hematology, and emergency surgical. A CRBSI risk of 1.78% in the 7-day group and 1.46% in the standard 4-day group was reported. The 7-day strategy was associated with annual savings of approximately 52 million Australian dollars (A$) (95% uncertainty interval [UI], −A$42 841 427 to A$181 823 300) from fewer set changes, offset by A$3.1 million (95% UI, −A$6 974 903 to A$14 099 754) in additional costs for treating CRBSI. The expected health outcomes were 395 (95% UI, −945 to 1739) additional infections, 103 (95% UI, −246 to 452) excess deaths, and 1724 (95% UI, −4199 to 7925) life-years lost at the population level. At a willingness-to-pay threshold of A$28 033, the probability that the 7-day strategy is cost-effective was 50.3%, and the probability that it is cost-saving was 82.67%.

**Conclusions and Relevance:**

In this economic evaluation of the RSVP trial, a small increase in CRBSI risk was associated with large cost-savings and substantial health losses at the population level. While the RSVP trial assumed that a 2% equivalence margin was acceptable, the use of such arbitrary difference margins might not consider information that could change decision-making outcomes. The clinical and economic impacts of small increases to infections appear to be important for decision-making inference.

## Key Points

**Question:**

Is adopting a 7-day infusion set replacement interval for catheter-related bloodstream infections cost-effective, without increasing infection risk, compared with the standard practice of 4-day replacement intervals?

**Findings:**

In this economic evaluation including 2941 patients in the RSVP (Replacement at Standard vs Prolonged Interval) trial, replacing infusion sets at 7 instead of 4 days was associated with reduced costs but increased risk of catheter-related bloodstream infections. There was a 50.3% likelihood of cost-effectiveness and 72.5% likelihood of worse health outcomes.

**Meaning:**

Results of this study suggest that there is a need for full cost-effectiveness analyses to assess the trade-off between the changes to total costs and health outcomes.

## Teaser

This economic evaluation study assesses whether adoption of a 7-day vs 4-day infusion set replacement interval for catheter-related bloodstream infection is cost-effective and whether there are health trade-offs in patients from the RSVP (Replacement at Standard vs Prolonged Interval) trial.

## Introduction

Catheter-related bloodstream infections (CRBSIs) are nosocomial infections related to the insertion of central venous access devices or peripheral arterial catheters. CRBSIs can cause substantial harm for patients^1^ and have large cost impacts due to prolongation of length of stay in hospital and from extra treatments.^2^ The Replacement at Standard vs Prolonged Interval (RSVP) trial is a large multicenter randomized clinical trial, which investigated whether extending infusion set replacement intervals from 4 to 7 days achieved CRBSI equivalence while assessing changes to costs.^3^ Within the trial, changes to CRBSI within a 2% margin were deemed equivalent between the groups. Changes in costs related to insertion and management of lines were estimated from the difference of costs incurred for the comparator groups. The trial did not consider changes in costs related to treatments or changes in patient outcomes arising from CRBSI.

Between 2012 and 2016, patients in the RSVP trial were randomized to 4-day vs 7-day changes of infusion sets. The trial revealed that, for patients with a central venous access device, 1.78% in the 7-day and 1.46% in the 4-day group acquired a CRBSI. The absolute risk difference was 0.32% (95% CI, −0.73%, 1.37%), which was within the 2% equivalence margin, and the hypothesis of equivalence was accepted.^3^ The cost analysis found an 89% probability of reduced costs in the 7-day compared with the 4-day group, when costs related to insertion and management of lines were tabulated.^3^ Based on these findings, the authors concluded that extending replacement intervals from 4 to 7 days for central venous access devices was a safe decision.

Given the millions of central and arterial vascular catheters placed each year worldwide and the associated negative impact of bloodstream infections, the findings from the RSVP trial are important.^4^ However, the trial did not consider the changes in treatment costs or health effects arising from the modest increase in CRBSI risk from the 7-day replacement, which occurs within the 2% equivalence margin. A complete economic evaluation that considers these aspects would provide valuable information on whether the 7-day replacement intervals are acceptable as a new policy to be recommended compared with the current 4-day standard of practice. The aim of our study was to estimate the changes to total cost and health benefits measured by life-years from a decision to adopt 7-day over 4-day infusion set replacement intervals. We report on the cost-effectiveness of a decision to adopt 7-day intervals from the health system perspective using data collected from the RSVP trial and from published literature.

## Methods

### Population and Setting

The RSVP trial was conducted from 2011 to 2016 across 10 Australian hospitals.^3^ The protocol^5^ for the RSVP trial and the findings^3^ have been published. The study enrolled 2944 hospitalized adults and children who required either central venous access devices or peripheral arterial catheters for at least 7 days. Patients were excluded if their device had been in situ for more than 96 hours, if they had a bloodstream infection within the previous 48 hours, or if the original infusion set had been replaced. The population sample randomized for the RSVP trial comprised 62.9% males and 62.6% who were admitted to the intensive care unit (ICU) during their admission. The patients were admitted to these clinical specialties: medical (32.0%), hematology (22.0%), emergency surgical (16.0%), elective surgical (10.0%), cardiac surgical (8.0%), trauma (7.0%), oncology (4.5%), and burns (1.0%).^3^ Data were analyzed from December 12, 2016, to April 23, 2019. This cost-effectiveness study used only data from published sources and therefore did not require institutional review board approval or informed consent according to the National University of Singapore Institutional Review Board's Review Not Required policy.

### Study Design, Perspective, and Comparators

**Figure 1.**
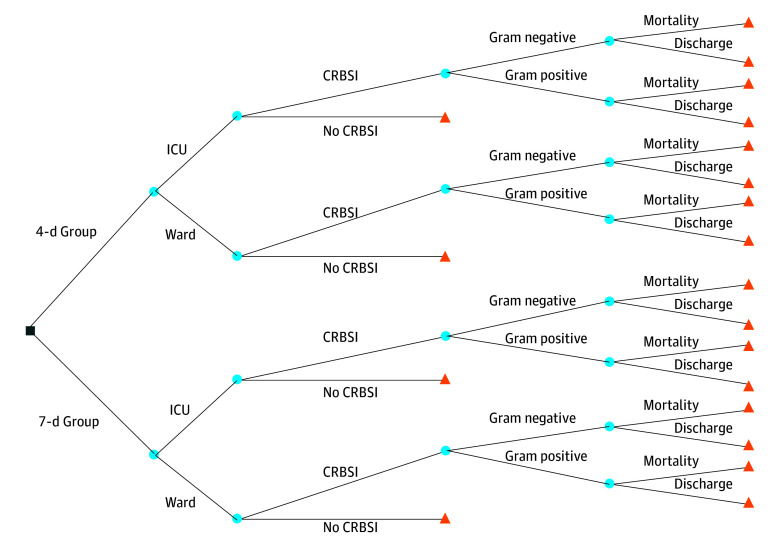
Decision Analytic Model Tree Diagram The square indicates a decision node. Blue circles indicate chance nodes, and orange triangles indicate terminal nodes. CRBSI indicates catheter-related bloodstream infection; ICU, intensive care unit.

A decision analytic model was developed on August 26, 2025, for this study to capture the competing risks of patients being infected, discharged, or dying (Figure 1). A patient’s probability of being admitted to a ward or ICU, developing a gram-positive or gram-negative CRBSI, and dying or being discharged is modeled, where ICU admission and gram-negative CRBSI are associated with greater costs.^6^ The perspective for this study is that of the hospital, as most costs incurred from infusion set changes and the consequences of CRBSI arise in that setting. The measured outcomes are used to inform the incremental change to total costs and life-years from a decision to extend the duration of infusion set replacements from 4 to 7 days for patients who receive a central venous access device in an Australian acute care public hospital. We excluded data on peripheral arterial catheters for 2 reasons: first, the RSVP trial evaluated them using a noninferiority design, which differed from the equivalence framework used for central venous access devices; and second, due to the low CRBSI rate for peripheral access devices (1 of 720 devices), which would result in uncertain or negligible outcomes. The economic evaluation adhered to the International Society for Pharmacoeconomics and Outcomes Research (ISPOR) reporting guideline, and the cost-effectiveness analysis using clinical trial data adhered to the relevant portions of the Consolidated Health Economic Evaluation Reporting Standards (CHEERS) reporting guideline.^7,8^

### Effectiveness, Health Outcomes, and Cost Measurement

Health benefits are shown by mortality changes arising from CRBSI expressed as life-years. All costs are reported in 2016 Australian dollars (A$) (2016 A$1 = US dollars $0.7438).^9^ The outcomes included in this cost-effectiveness analysis were the number of patients who developed CRBSI, the number of hospital and ICU bed-days used for patients with CRBSI, the cost of hospital and ICU bed-days used, the number of deaths caused by CRBSI, and the number of discounted life-years lost. Parameters used to inform the model included the cost of a hospital bed-day,^10^ life expectancy of the Australian population,^11^ and the probability of mortality from CRBSI.

Patients were followed up until catheter removal, discharge with catheter in situ, or 21 days after the last infusion, which allows for the capture of CRBSI and resource use during the hospital stay. To capture longer-term costs and health outcomes, we used external data sources to inform the length of stay and mortality estimates associated with CRBSI.^6,12^ These studies were conducted in the Australian context and are reflective of the local impact of CRBSI on patients. Where relevant data were unavailable, we undertook a meta-analysis to inform model parameters^13^ (Table 1). The meta-analysis was used to derive mortality outcomes attributable to CRBSI, as such estimates were not available from the RSVP trial or from published literature. The authors of the RSVP trial excluded 3 patients from the analysis as 1 patient had no central venous access device, 1 patient received no intervention, and 1 patient withdrew, which left 2941 patients who were included for analysis.

**Table 1.** Parameters Used to Inform Outcomes and Cost-Effectiveness From Meta-Analysis

Parameter	Source	Distribution^a^	Estimate (distribution parameter α, β)
Probability of CRBSI			
4-d Interval group	Rickard et al,^3^ 2021	Beta	0.0145 (α, 16; β, 1081)
7-d Interval group			0.0176 (α, 20; β, 1104)
Probability of gram-negative CRBSI			
4-d Interval group	Rickard et al,^3^ 2021	Beta	0.56 (α, 9; β, 7)
7-d Interval group			0.19 (α, 4; β, 17)
Probability of being in the ICU			
4-d Interval group	Rickard et al,^3^ 2021	Beta	0.49 (α, 513; β, 514)
7-d Interval group			0.47 (α, 508; β, 551)
Infusion set replacement cost, AUD (SD)^b^			
4-d Interval group	Rickard et al,^3^ 2021	Gamma	823 (420)
7-d Interval group			339 (168)
Length of stay, d, mean (SD)			
Excess hospitalization LOS for gram-positive BSI	Barnett et al,^6^ 2013	Gamma	9.8 (1.25)
Excess hospitalization LOS for gram-negative BSI			2.7 (2.60)
Excess ICU LOS for gram-positive BSI			0.9 (0.36)
Excess ICU LOS for gram-negative BSI			0.6 (0.18)
Other parameters			
Cost of ward bed-day, AUD^b^	AIHW,^10^ 2017	Fixed	1667
Cost of ICU bed-day, AUD^b^			6280
2021 Australian life expectancy	ONS,^11^ 2020	Fixed	83.3 y
Discounted CRBSI LYG, y, mean (SD)	Rickard et al,^3^ 2021	Gamma	16.8 (3.28)
Central venous access devices, No.	Tuffaha et al,^12^ 2019	Fixed	125 710
Probability of mortality attributable to CRBSI	Elangovan et al,^13^ 2024	Beta	0.260 (α, 245; β, 695)

Abbreviations: AIHW, Australian Institute of Health and Welfare; AUD, Australian dollar; BSI, bloodstream infection; CRBSI, catheter-related bloodstream infection; ICU, intensive care unit; LOS, length of stay; LYG, life-years gained; ONS, Office of National Statistics.

^a^ Gamma distributions were used to model cost and time parameters to reflect positive skew, while probabilities with values between 0 and 1 were modeled with beta distributions. Fixed values indicate that a single point estimate was used. Uncertainties were summarized by 10 000 random samples from the prior distributions.

^b^ AUD $1 = USD $0.7438.

#### Number of Patients Likely to Receive a Central Venous Access Device Annually

There are 26 399 central venous access devices placed annually in public hospital patients in Queensland,^12^ 1 of 6 states in Australia. Queensland is responsible for 19.87% of all episodes of acute inpatient care in Australian public hospitals that involved at least 1 overnight stay.^14^ From this, we estimated the number of central venous access devices placed in Australia to be 125 710 per year (Table 1).

#### Change to Costs Directly Arising From Infusion Set Changes

Information about nursing time required to replace infusion sets, the associated staffing costs, and the costs of consumables have been reported.^3^ Nursing time was calculated from a time-in-motion study during the trial and employment costs were based on employment awards for nurses.^3^ Consumables used and the prices paid were obtained from the procurement unit of the hospital during the trial. This information was combined with data on the number of replacements for patients reported in each arm of the trial. The mean (SD) cost of time and consumables per patient of managing central venous access devices was $339 ($168) for the 7-day group and $823 ($420) for the 4-day group.

#### Difference in the Number of Cases of CRBSI

The RSVP data were used to estimate the probability of CRBSI under the conditions of 7-day and 4-day intervals (Table 1). A cumulative incidence risk was estimated from the events observed during the trial. In the 4-day arm, there were 16 cases of CRBSI observed in 1097 patients; in the 7-day arm, there were 20 cases observed in 1124 patients.

#### Attributable Bed-Days Used to Treat CRBSI and Increased Risk of Death

The RSVP trial was not designed to estimate either of the key outcomes of attributable bed-days used to treat CRBSI and increased risk of death, so other information sources were used.^6^ Data were made available for all patients admitted to the 9 largest public hospitals in Queensland between January 1, 2005, and December 31, 2010. All cases of CRBSI were classified by type, which overlapped with the groups reported in the RSVP trial—that is, CRBSI with gram-positive and gram-negative bacteria. The estimates of prolongation of lengths of stay and relative risks of mortality for all relevant bloodstream infections are shown in Table 1. Changes in the number of deaths from the adoption of 7-day intervals were estimated by considering the difference in the number of infections and then applying the risk of death for uninfected and the relative risk of death given infection. Estimates of a meta-analysis of in-hospital all-cause mortality were used to inform the increased risk of death due to CRBSI.^13^

#### Monetary Cost of Bed-Days

Monetary values for both ward and ICU bed-days were derived from an accounting method that divided total recurrent expenditure on admitted care by the total number of patient-days in Australian public hospitals in 2016.^15^ An accounting model likely overvalues the cost of a lost bed-day compared with other approaches.^16,17,18^ Using an accounting model is a conservative way to examine cost-effectiveness in this context.

#### Changes in Life-Years

The median age of the RSVP trial participants was 59.0 years (range, 47-68 years) and their life expectancy was 83.3 years.^19^ Future years of life saved are discounted at 3%.

### Accounting for Uncertainty and Cost-Effectiveness Threshold

We estimated statistical distributions or used fixed values for a range of relevant parameters that inform the model. Gamma distributions were used to model cost and time parameters to reflect positive skew, while probabilities with values between 0 and 1 were modeled with beta distributions. Fixed values indicate that a single point estimate was used. Uncertainties were summarized by 10 000 random samples from the prior distributions (Table 1). This propagates forward all uncertainties, and outcomes are shown by the joint distribution of the change to total costs and change to health benefits on a cost-effectiveness plane. Due to the acute nature of CRBSI, where impacts on health-related quality of life are short-lived, we assumed that quality-adjusted life-years and life-years gained are equivalent as change is likely related to life-years and not quality of life. The proportion of resamples that fall below the willingness-to-pay threshold of $28 033 per life-year gained^20^ will indicate the probability in which adopting 7-day replacement intervals is cost-effective. A scenario analysis was conducted to reflect a risk difference of 1.99% between the 4-day and 7-day replacement intervals, compared with the observed risk difference of 0.32%. We used a risk difference of 1.99% because it reflects the largest value that is considered to be acceptable within the 2% equivalence margin defined in the RSVP trial.^3^

### Statistical Analysis

To evaluate decision uncertainty across varying willingness-to-pay thresholds, we constructed a cost-effectiveness acceptability curve. Net monetary benefit was calculated for both replacement intervals across willingness-to-pay values ranging from $0 to $100 000 in $1000 increments. At each threshold, we computed the proportion of simulations in which the 7-day interval had the higher net monetary benefit, representing the probability that it is cost-effective. We conducted a value of information analysis using the BCEA software and voi packages in R, version 4.5.1 (R Foundation for Statistical Computing). To quantify the value of reducing decision uncertainty, we estimated the expected value of perfect information across a willingness-to-pay range of $0 to $100 000 in $1000 increments. The expected value of partial perfect information was calculated, and key drivers of uncertainty and parameters with the highest values are reported.

## Results

We observed cost-savings from the 7-day interval of approximately $52 million per year (95% uncertainty interval [UI], -$42 841 427 to $181 823 300) accompanied by 395 (95% UI, −945 to 1739) additional cases of CRBSI that used 1409 (95% UI, −2649 to 6125) ward and 121 (95% UI, −450 to 756) ICU bed-days (Table 2). These additional bed-days incurred accounting costs of approximately $3.1 million (95% UI, -$6 974 903 to $14 099 754). While costs related to additional CRBSIs were low relative to the cost-savings of the 7-day replacement interval, the associated health outcomes were more consequential, with 103 (95% UI, −246 to 452) additional deaths and 1724 (95% UI, −4199 to 7925) life-years lost.

**Table 2.** Population-Level Incremental Changes in Outcomes From Adopting 7-Day vs 4-Day Infusion Set Replacement Intervals

Outcome	Mean incremental change (95% UI)
Costs from fewer set changes, AUD^a^	−51 730 024 (−42 841 427 to 181 823 300)
Cases of CRBSI, No.	395 (−945 to 1739)
Ward bed-days used	1409 (−2649 to 6125)
ICU bed-days used	121 (−450 to 756)
Costs from all bed-days used, AUD^a^	3 110 049 (−6 974 903 to 14 099 754)
Deaths, No.	103 (−246 to 452)
Life-years	−1724 (−4199 to 7925)

Abbreviations: AUD, Australian dollar; CRBSI, catheter-related bloodstream infection; ICU, intensive care unit; UI, uncertainty interval.

^a^ AUD $1 = USD $0.7438.

**Figure 2.**
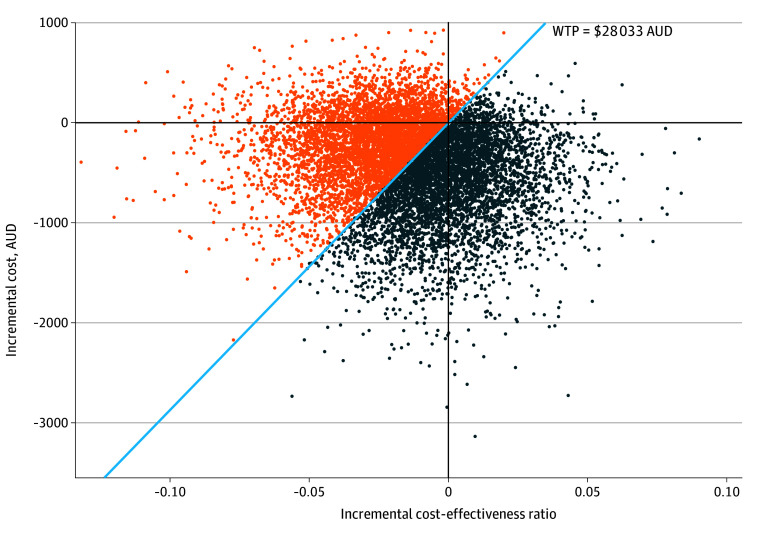
Joint Distribution of Change to Total Costs and Change to Health Benefits Dark blue circles indicate simulations where the 7-day strategy is cost-effective; orange circles indicate simulations where it is not cost-effective. AUD $1 = USD $0.7438. AUD indicates Australian dollar; WTP, willingness-to-pay.

The joint distribution of the change to total costs and change to life-years is summarized in Figure 2. We included a willingness-to-pay threshold of A$28 033 per life-year. A result below this threshold suggests that adoption is cost-effective.^21^ We therefore consider the 5032 of the 10 000 simulated results below that level as a 50.32% probability that adoption of the 7-day interval is cost-effective and a 49.68% probability that adoption is not cost-effective. In most simulations, there was a net loss in health outcomes due to small increases in CRBSI-related mortality. The 7250 results associated with negative incremental effectiveness indicate that, in 72.50% of simulations, the 7-day interval results in fewer life-years than the 4-day interval. Additionally, 8267 out of 10 000 results were associated with negative incremental costs, showing 82.67% probability that adoption will be cost-saving.

### Scenario Analysis

In the scenario analysis, a range of risk differences from 0.01% to 1.99% was used to estimate associated outcomes (Table 3). Under the assumption of a risk difference of 1.99%, the model predicted 2502 additional cases of CRBSI, 651 excess deaths, and 10 931 years of life lost with the 7-day strategy. The probability of cost-effectiveness was estimated to be less than 1%.

**Table 3.** Scenario Analysis of Cost-Effectiveness and Outcomes Associated With Range of Risk Differences

Risk difference, %	7-d Strategy			
	Probability of cost-effectiveness, %	Additional cases of CRBSI	Additional deaths	Additional life-years lost
0.01	84.62	13	3	54.9
0.1	74.78	126	33	549.3
0.2	62.50	251	65	1098.6
0.3	50.07	377	98	1647.9
0.4	38.17	503	131	2197.2
0.6	20.45	754	196	3295.8
0.8	10.29	1006	262	4394.4
1.2	2.49	1509	392	6591.5
1.6	0.61	2011	523	8788.7
1.99	0.12	2502	651	10 931.0

Abbreviation: CRBSI, catheter-related bloodstream infection.

### Cost-Effectiveness Across Willingness-to-Pay Thresholds

The cost-effectiveness acceptability curve illustrates the probability that each replacement interval is cost-effective across a range of willingness-to-pay thresholds (eFigure 1 in Supplement 1). At lower thresholds, the 7-day interval had a higher probability of being cost-effective, which decreased as the threshold increased, reaching a parity threshold of $28 033 used in this study. The 7-day interval also failed to have a higher probability of cost-effectiveness than the 4-day interval at any willingness-to-pay threshold above $28 000.

### Value of Information Analysis

The expected value of perfect information at the threshold of $28 033 was $327.12 per patient, representing the maximum value of eliminating uncertainty across all parameters (eFigure 2 in Supplement 1), which increased with threshold values up to $100 000 (eFigure 3 in Supplement 1). The parameters with the largest expected value of partial perfect information parameters were the probabilities of CRBSI followed by the cost parameters for both interval groups.

## Discussion

There is weak evidence to support the conclusion that adoption of a 7-day infusion set replacement interval is cost-effective. This emerges from findings that adoption could reduce health benefits while reducing costs, compared with the 4-day replacements. There are considerable uncertainties and low confidence about the conclusion of the RSVP trial that extending replacement intervals is a good decision for health services.^3^

The tension between the conclusions from the RSVP trial and this cost-effectiveness analysis arises because of the simplifying assumption that a difference of less than 2% in the primary outcome of CRBSI did not matter. While a 2% noninferiority margin may appear small, we showed that an absolute risk difference of 0.32% will already likely lead to 395 extra cases of CRBSI, 103 deaths, and 1724 years of life lost when scaled to the population level. Our scenario analysis further showed that, if the absolute risk difference had been 1.99%, equivalence would still have been concluded from the trial, yet the cost and health impacts would be strongly negative. This suggests that, in this specific context, the arbitrarily chosen 2% margin may have been too permissive, and earlier consideration of the downstream health losses could have allowed a lower and more appropriate margin to have been chosen. Alternatively, a risk-stratified approach to determining replacement intervals, based on CRBSI risk, could have been adopted to mitigate the expected poor health outcomes.^22,23^

Our study found that there is some evidence to support the cost-effectiveness of the intervention despite reduced health benefits, because it saves resources or costs of approximately $53 million per year. This large cost-savings is just sufficient to offset the reductions in health benefits and meet the criterion of cost-effectiveness. The results of our analysis are useful because parameters were specified from high-quality data sources and capture costs associated with managing CRBSIs. Our value of information analysis highlights uncertainty in key parameters, particularly the probabilities of CRBSI and associated costs for both 4-day and 7-day intervals. Future research efforts should prioritize these parameters to strengthen the evidence base for changes to infusion set replacement intervals.

### Limitations

This study has some limitations. First, these findings are based on Australian data sources. Variation in CRBSI rates or hospital costs may alter cost-savings and health outcomes, warranting local cost-effectiveness evaluation.^24^ Second, we assumed that quality-adjusted life-years were equivalent to life-years gained to reflect the short-term effects of CRBSI. However, this simplified assumption does not account for any morbidity or health-related quality-of-life impacts following CRBSI, and future work should incorporate utility estimates where available. Last, this study used a decision-analytic framework, which inherently simplifies the progression of CRBSI-associated events.^25^ More complex approaches, such as individual patient simulations or dynamic models, could provide a better representation of CRBSI pathways.

## Conclusions

Findings of this study suggest that basing policy decision on arbitrary definitions of equivalence that appear reasonable statistically may fail to capture health and economic consequences of adoption. While the interpretation of the RSVP trial supported adoption for 7-day intervals, the analysis was done on selected acute outcomes and did not account for the downstream health and economic consequences of increased CRBSI risk. For decision-making and given the implications of the RSVP trial findings worldwide, we believe it is important to undertake cost-effectiveness analyses that include a wider range of outcomes, consider the impact of effect sizes, and report uncertainty for decision-making. Although the RSVP trial was used as the basis of this study, the findings highlight the need for early trial-based economic evaluations to ensure that margin choices and outcome measures adequately reflect both clinical and economic consequences.

## Notes

Supplement 1. **eFigure 1.** Cost-effectiveness acceptability curve across willing-to-pay thresholds **eFigure 2.** Value of Information analysis **eFigure 3.** Expected Value of Perfect Information across willingness-to-pay thresholds

Supplement 2.
Data Sharing Statement
