# Supplementary material for: Seven-Day vs Four-Day Infusion Set Replacement Interval and Catheter-Related Infections
Source: JAMA Netw Open. 2025 Dec 2;8(12):e2546398. doi: 10.1001/jamanetworkopen.2025.46398 (PMC12673407; doi:10.1001/jamanetworkopen.2025.46398)
Supplement: Supplement 1. — eFigure 1. Cost-effectiveness acceptability curve across willing-to-pay thresholds eFigure 2. Value of Information analysis eFigure 3. Expected Value of Perfect Information across willingness-to-pay thresholds [file jamanetwopen-e2546398-s001.pdf]

Supplemental Online Content

Elangovan S, Cai Y, Mitchell BG, Graves N. Seven-day vs four-day infusion set replacement interval and catheter-related infections. *JAMA Netw Open*. 2025;8(12):e2546398. doi:10.1001/jamanetworkopen.2025.46398

eFigure 1. Cost-effectiveness acceptability curve across willing-to-pay thresholds
eFigure 2. Value of Information analysis
eFigure 3. Expected Value of Perfect Information across willingness-to-pay thresholds

This supplemental material has been provided by the authors to give readers additional information about their work.

© 2025 Elangovan S et al. *JAMA Network Open*.

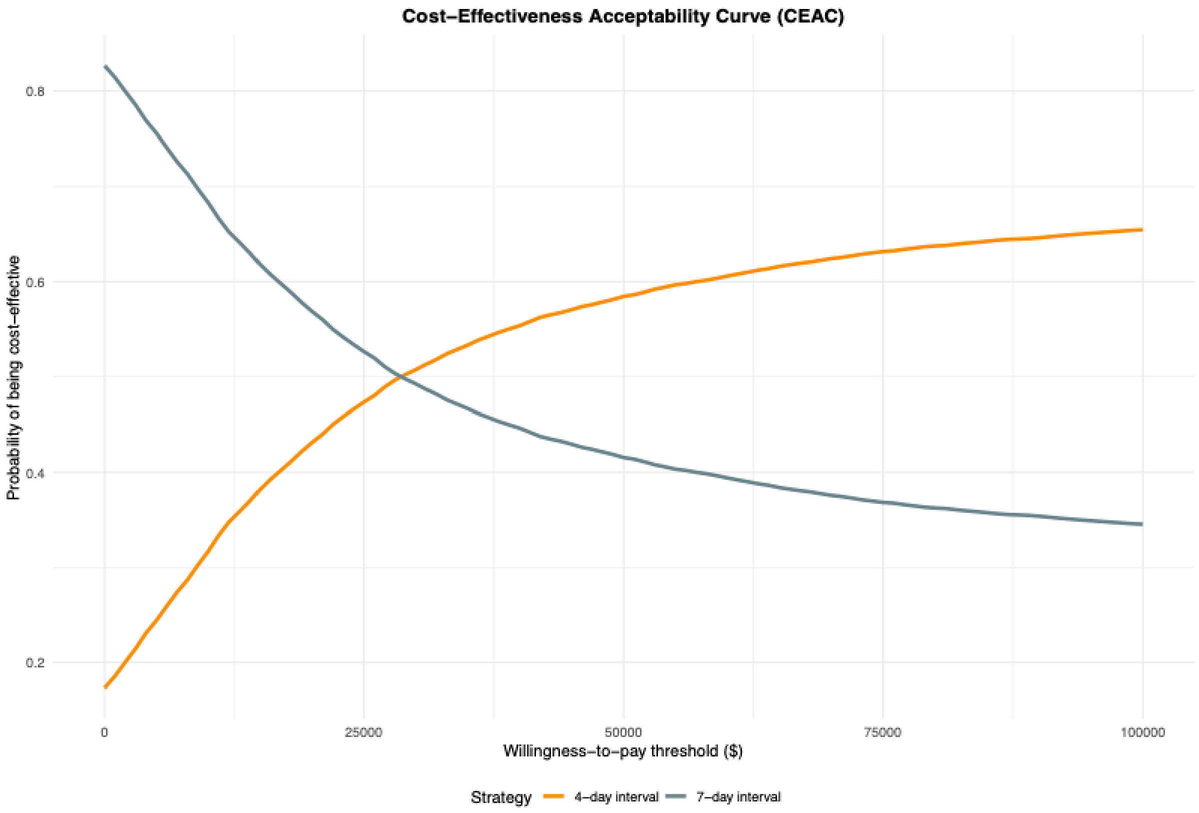

eFigure 1 Cost-effectiveness acceptability curve across willing-to-pay thresholds

© 2025 Elangovan S et al. *JAMA Network Open*.

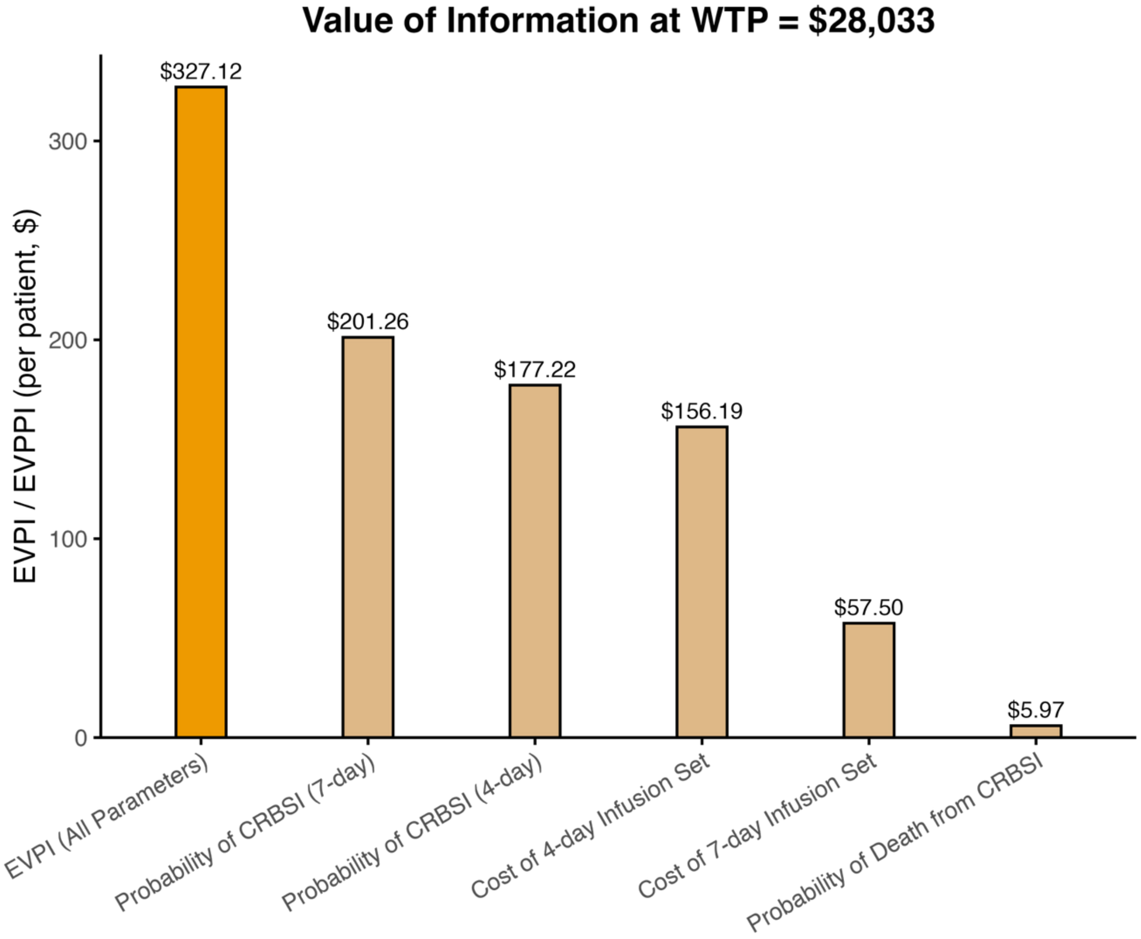

eFigure 2 Value of Information analysis

© 2025 Elangovan S et al. *JAMA Network Open*.

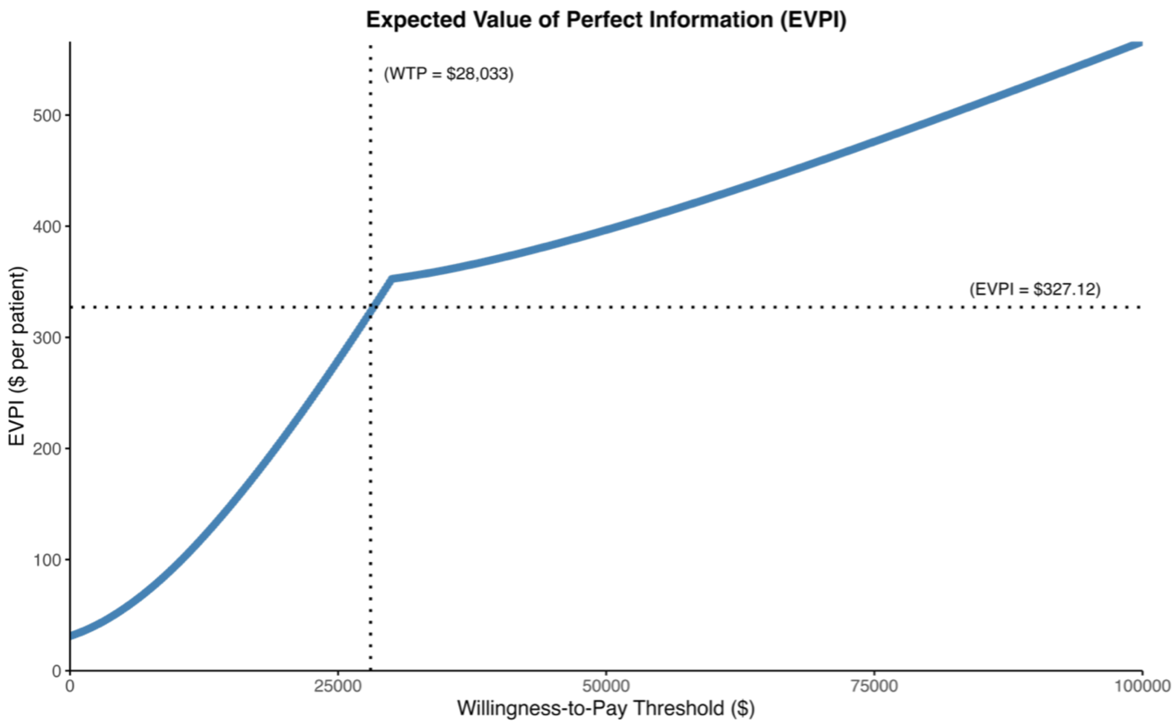

eFigure 3 Expected Value of Perfect Information across willingness-to-pay thresholds

© 2025 Elangovan S et al. *JAMA Network Open*.
