# Supplementary material for: Seven-Day vs Four-Day Infusion Set Replacement Interval and Catheter-Related Infections
Source: JAMA Netw Open. 2025 Dec 2;8(12):e2546398. doi: 10.1001/jamanetworkopen.2025.46398 (PMC12673407; doi:10.1001/jamanetworkopen.2025.46398)
Supplement: Supplement 2. — Data Sharing Statement [file jamanetwopen-e2546398-s002.pdf]

Data Sharing Statement

Elangovan. Seven-Day vs Four-Day Infusion Set Replacement Interval and Catheter-Related Infections. *JAMA Netw Open*. Published December 02, 2025.
doi:10.1001/jamanetworkopen.2025.46398

Data
Data available: No

Additional Information
Explanation for why data not available: There is no primary data collection associated with this study.
